# Protein Signature in Saliva of Temporomandibular Disorders Myalgia

**DOI:** 10.3390/ijms21072569

**Published:** 2020-04-07

**Authors:** Hajer Jasim, Malin Ernberg, Anders Carlsson, Björn Gerdle, Bijar Ghafouri

**Affiliations:** 1 Division of Oral Diagnostics & Rehabilitation, Department of Dental Medicine, Karolinska Institutet and Scandinavian Center for Orofacial neuroscience (SCON), SE 14104 Huddinge, Sweden; 2 Pain and Rehabilitation Centre, and Department of Health, Medicine and Caring Sciences, Linköping University, SE 581 83 Linköping, Sweden

**Keywords:** chronic pain, proteomics, saliva, temporomandibular disorders

## Abstract

In the last years, several attempts have been made to study specific biological markers of temporomandibular disorders (TMD). So far, no laboratory tests have been appropriately validated for the diagnosis and prognosis of these disorders. This study aimed to investigate the proteomic profile of the whole stimulated saliva of TMD myalgia patients in order to evaluate potential diagnostic and/or prognostic salivary candidate proteins which could be useful for the management of TMD. Twenty patients diagnosed with TMD myalgia according to the validated Diagnostic Criteria for TMD (DC/TMD) and 20 matched healthy pain-free controls were enrolled. Saliva samples were collected in the morning. Comparative proteomic analysis was performed with two-dimensional gel electrophoresis followed by identification with liquid chromatography–tandem mass spectrometry. Statistical analysis of the quantitative proteomics data revealed that 20 proteins were significantly altered in patients compared to controls. Among these proteins, 12 showed significantly increased levels, and 8 showed significantly decreased levels in patients with TMD myalgia compared to controls. The identified proteins are involved in metabolic processes, immune response, and stress response. This proteomic study shows that the salivary protein profile can discriminate patients with TMD myalgia from healthy subjects, but the protein signature has no correlation with the clinical features of TMD myalgia. Additional studies are needed to validate our observations in additional sample sets and to continue assessing the utility of saliva as a suitable sample for studying processes related to TMD myalgia.

## 1. Introduction

Temporomandibular disorders (TMD) are a cluster of conditions that cause pain and dysfunction in the temporomandibular joint, masticatory muscles, and surrounding structures, e.g., ligaments and connective tissues [1,2]. It causes high suffering to those affected in the community and is a widespread problem in clinical practice [2,3,4,5]. TMD affects 10–15% of the adult population and seems to be three times more frequent in women [1,2]. TMD pain of muscular origin, e.g., TMD myalgia, is the most common diagnosis, with a frequency of 42% [2]. This pain condition affects the quality of life considerably and is the major cause of non-odontogenic pain [2,3,4]. Clinically, there are subclasses of myalgia, but all are based on similar diagnostic criteria. These subclasses differ only for the presence of pain distribution upon palpation. However, the pathogenesis underlying these subclasses may not be the same [6].

The causes of TMD myalgia are complex and multifactorial and involve a combination of psychological, physiological, structural, postural, and genetic factors [2,3,4,5]. The pathophysiological mechanisms behind TMD pain are poorly understood, which requires the healthcare profession to purely rely on patient history, questionnaires, and semi-objective findings for diagnostic and prognostic purposes. Pain is, however, a subjective experience, and existing methods like pain drawings, muscle palpation, or assessment of pain threshold have limited sensitivity and correlate weakly with ongoing pain intensity. Hence, there is a need for more objective and sensitive methods [7]. 

Proteomics, defined as the systematic analysis of proteins expressed by an organism at a given time, under certain conditions, has become a powerful tool in bioscience to identify new disease-specific proteins [8,9,10,11]. Several proteomic studies have been successfully performed for different painful condition, e.g., rheumatoid arthritis, neuropathic pain, fibromyalgia, burning mouth syndrome, and trapezius myalgia by analyzing proteins in saliva, plasma, synovial fluid, interstitial fluid, or biopsies [12,13,14,15,16,17,18,19,20,21]. 

Saliva is an outstanding body fluid containing a complex mixture of proteins, peptides, and other substances that may yield information about the pathophysiology of TMD myalgia and can be used to identify new candidate proteins of the disorder. Proteomic analysis of saliva from patients with TMD myalgia represents a new potential field of research, as the proteomic techniques are constantly improving [22,23,24]. Two studies, so far, have investigated the salivary proteome of patients with widespread myalgia. The authors applied gel-based proteomics to saliva samples from patients with fibromyalgia and reported altered protein expression between patients and controls, with an over-expression of transaldolase, phosphoglycerate mutase I, serotransferrin, and alpha-enolase. However, none of these candidate proteins showed a correlation with clinical features [13,21].

The aim of this study was therefore to compare the global protein profile of saliva between patients with TMD myalgia and age- and sex-matched controls to search for potential diagnostic or prognostic salivary candidate proteins for TMD myalgia.

## 2. Results

### 2.1. Clinical Outcomes

The descriptive data of all patients and healthy controls in the study are presented in Table 1. Patients and controls were similar in background factors, such as country of birth, occupation, education level, and level of physical activity. Patients included in the study showed significantly higher signs of psychological distress and decreased pain-free jaw opening compared to controls. Patients expressed on average mild depressive symptoms and tendency towards insomnia, moderate levels of somatic symptoms and perceived stress, and almost no pain catastrophizing. The patients reported an average pain duration (± standard deviation, SD) of 6.3 ± 6.3 years and a median (interquartile range, IQR) characteristic pain intensity (CPI) of 65 (27). The median current pain intensity on a 0–10 numeric rating scale (NRS) was 6 (3). Patients with TMD myalgia could further be separated in subclasses based on the main diagnosis according to the Diagnostic Criteria for TMD (DC/TMD). Ten patients were diagnosed with myalgia and 10 with myofascial pain (with or without referral). When further investigating these groups, some dissimilarities emerged; patients with myofascial pain showed a significantly longer duration of headache (*p* = 0.037) and reported higher current pain intensity (*p* = 0.023) and CPI (*p* = 0.023) than patients with myalgia. However, they showed lower physical activity level (*p* = 0.039) and lower pressure pain threshold (PPT) of the masseter muscle (*p* = 0.001) compared to patients with myalgia.

### 2.2. Proteome Pattern in TMD Myalgia

Twenty patient samples and 20 control samples were chosen for comparative proteomic analysis. The patients were well matched in terms of age, gender, and demographic variables to reduce bias from these factors during the discovery stage. Total protein concentration was measured prior to two-dimensional gel electrophoresis (2-DE) analysis, and equal amount of protein from each sample was used for the analysis. The median values of total protein concentration were 2.84 µg/µl for the patient group and 3.3 µg/µl for the control group. There were no statistically significant differences between the groups (*p* = 0.52). Further, there was no statistically significant difference in salivary flow rate between the groups (Table 1). A total of 197 protein spots were matched and included in the statistical analysis.

**Figure 1:**
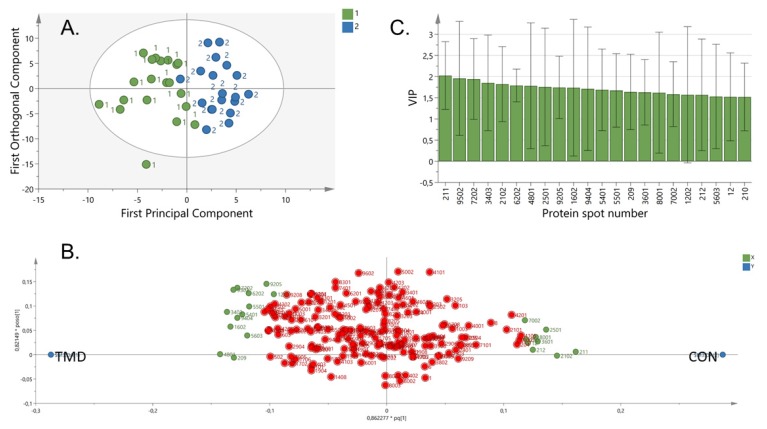
**Multivariate analysis.** Graphs showing proteins that differed between 20 patients with temporomandibular disorders (TMD) myalgia and 20 age- and sex-matched controls (CON). (**A**). orthogonal partial least-square discriminant analysis models showing separation between patients with TMD (green circle marked as number 1) and healthy controls (blue circle marked as 2). The longitudinal dimension (y-axis) shows the interclass discrimination, and the latitudinal dimension (x-axis) shows the intraclass discrimination between the groups. (**B**). Loading score highlighting proteins of importance for the separation. Green circles refer to proteins with variable importance of projection (VIP) > 1.5. Proteins on the right are positively associated with CON, and those on the left are positively associated with TMD. (**C**). VIP values for proteins. VIP values > 1.5 were considered significant. Protein spot numbers 211 and 9502 were the most important proteins for group separation (TMD vs. CON).

**Figure 2:**
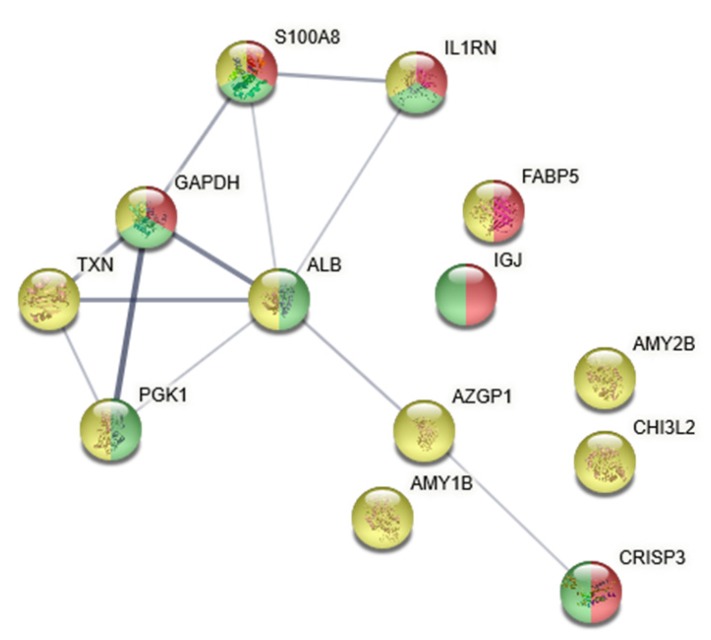
**Network analysis.** Network analysis of important proteins for separating patients from controls using the Search Tool for Retrieval of Interacting Genes/Proteins (STRING) data analysis. The line thickness indicates the strength of data support. The majority of proteins were related to the regulation of response to metabolic processes, with a false discovery rate (FDR) of 0.03 (highlighted in yellow), to the response to stress (FDR of 0.02, highlighted in green), and to the immune response (FDR of 0.01, highlighted in red). The nodes are marked with the gene names of the proteins, and the corresponding protein names were: GAPDH = glyceraldehyde-3-phosphate; TXN = thioredoxin; PGK1 = phosphoglycerate kinase 1; ALB = serum albumin; S100A8 = protein S100-A8; IL1RN = interleukin-1 receptor antagonist protein; FABP5 = fatty acid-binding protein; IGJ = immunoglobulin J chain; AXGP1 = zinc-alpha-2-glycoprotein; AMY1B = amylase, alpha 1B; AMY2B = amylase, alpha 2B; CHI3L2 = chitinase-3-like protein 2; CRISP3 = cysteine-rich secretory protein family.

Multivariate statistical analysis using orthogonal partial least-squares discriminant analysis (OPLS-DA) showed a distinct difference in the proteome profile between TMD and controls (model characteristics *R*^2^ = 0.70, *Q*^2^ = 0.27, cross-validated analysis of variance (CV-ANOVA) = 0.03), (Figure 1). Prior to the OPLS-DA, unsupervised principal component analysis (PCA) was performed to find outliers. The PCA detected one subject as outlier based on the score plots in combination with Hotelling’s T2 (identifies strong outliers) and distance to model in X-space (identifies moderate outliers). This subject was excluded from further analysis. The comparative proteomic analysis revealed that 20 proteins with variable importance of projection (VIP) > 1.5 were up- or downregulated in patients compared to controls (Table 2). Most of the proteins with VIP > 1.5 also differed significantly (*p* < 0.05) using Mann–Whitney univariate statistics. Among these proteins, 12 showed significantly higher levels, whereas the remaining 8 showed significantly lower levels in patients with TMD myalgia compared to controls (Table 2). Network analysis showed that these 20 proteins were involved in metabolic processes (*n* = 11), immune response (*n* = 6), and response to stress (*n* = 7), as shown in Figure 2. The enrichment indicated that the proteins were at least partially biologically interconnected as a group (*p* < 0.001).

### 2.3. Correlation Analysis between Altered Levels of Proteins and Clinical Parameters

The expression levels of the 20 altered proteins (Table 2) were investigated to correlate them with clinical outcomes in patients using univariate Spearman correlation analysis with Bonferroni correction (adjusted *p* < 0.001). No statistically significant correlations were observed between the altered proteins and any of the following clinical parameters of TMD myalgia or its subclasses: mouth opening (pain-free and maximum unassisted), pain duration, current pain on the NRS, CPI, Graded Chronic Pain Scale (GCPS), Patient Health Questionnaire (PHQ-9 and PHQ-15), Generalized Anxiety Disorder scale (GAD-7), Pain Catastrophizing Scale (PCS), Perceived Stress Scale-10 (PSS-10), Jaw Functional Limitation Scale (JFLS), Insomnia Severity Index (ISI), and PPT of the masseter muscle.

The altered proteins were analyzed together with the clinical parameters to identify any differences between patients diagnosed with myalgia and patients diagnosed with myofascial pain. A significant OPLS model was found (model characteristics R^2^ = 0.71, Q^2^ = 0.41, CV-ANOVA = 0.01). Phosphoglycerate kinase 1 was the most important protein (VIP > 2) for separation between the two sub-diagnoses (Table 3). 

## 3. Discussion

In the present study, we describe the protein profile of saliva from patients diagnosed with TMD myalgia in comparison with that from healthy controls in order to identify potential candidate protein markers of the disorder. Twenty proteins were found to be significantly altered in patients compared to controls. These identified proteins are involved in metabolic processes, immune response, and response to stress. Interestingly, there were significant differences in the expression of some of these proteins between subclasses of TMD myalgia.

Phosphoglycerate kinase 1 (PGK1), a glycolytic enzyme catalyzing the transformation of 3-phosphoglycerate into 2-phosphoglycerate, was the most important protein for separating patients and controls and, further, subclasses of TMD myalgia: patients with a diagnosis of myalgia expressed significantly more PGK1 in their saliva compared to patients with myofascial pain. PGK1 expression has previously been described in various malignancies and was recently demonstrated to correlate to poor prognosis in breast cancer [25]. High expression of PGK1 has also been described in synovial tissue and blood of patients with rheumatoid arthritis, suggesting the involvement of the enzyme in the inflammatory process and synovial hyperplasia [26]. Deficiency of PGK1 usually causes hemolytic anemia and neurological impairment and, in rare cases, also muscle weakness and cramping [27]. Over-expression of salivary PGK1 in TMD myalgia patients has never been described so far; however, the involvement of the enzyme in TMD remains unclear and needs further evaluation. Glyceraldehyde-3-phosphate dehydrogenase (GAPDH) is another glycolytic enzyme that was found significantly upregulated in TMD myalgia patients and, like PGK1, is overexpressed in various malignancies and correlates positively with tumor progression [28,29]. GAPDH has also been discussed in various neurodegenerative diseases [29]. Giusti and co-authors analyzed the proteome of whole saliva in patients with systemic sclerosis and observed elevated GAPDH in patients compared to controls [30]. Glycolytic enzymes such as PGK1 and GAPDH are usually found in the cytoplasm and released into the general circulation during pathological states that correlate with cell damage or apoptosis [29]. In this context, it may be hypothesized that conditions of oxidative stress involved in myalgia may increase the need of PGK1 and GAPDH [31].

Another relevant observation emerged from the data analysis is the significant altered levels of the digestive and antimicrobial enzyme salivary alpha-amylase (sAA) in TMD myalgia patients compared to controls. The sAA levels were also significantly increased in patients with myalgia compared to those with myofascial pain (Table 3). Numerous studies have suggested sAA as a potential marker for sympatho-adrenal medullary activity [32,33,34]. There is also evidence that sAA concentrations are predictive of plasma catecholamine levels [35] and furthermore can be used as a valid indicator for measuring stress [33,36,37]. SAA significantly separated patients from controls and further distinguished between subclasses of TMD myalgia in our study sample. Patients also reported significantly higher levels of perceived stress, but no significant correlation could be observed between sAA and subjective stress. Therefore, the validity of sAA as a marker remains debatable, since its levels not always correlate with sympathetic activation. Studies have shown that the levels of sAA increase in individuals during physical and psychological stress [34,38,39,40,41]. It is known that pain can act as a potential stressor and affects psychological as well as physiological systems [5]. Recent studies have proposed sAA as a candidate protein for the objective assessment of pain intensity [42,43]. Shirasaki and co-authors measured sAA in patients with chronic back pain and found a significant decrease in sAA after pain reduction by epidural blockage; similar findings were obtained for odontogenic pain [42,43]. However, several other studies could not identify a significant relationship between ongoing pain and sAA levels [36,44,45].

Cysteine-rich secretory protein 3 (CRISP 3) is another upregulated protein in TMD myalgia patients. This protein was originally discovered in human neutrophils but is widely distributed in exocrine glands (salivary glands, pancreas, and prostate) and has been detected in small amounts in the thymus, colon, ovary, and epididymis. This protein appears to be linked to innate immunity and inflammation [46] and has recently been suggested as a potential candidate protein for prostate cancer [47]. Lane and co-authors found significantly lower levels of CRISP-3 in the saliva from patients with Sjögren’s syndrome compared to heathy controls and concluded that the CRISP-3 deficiency in Sjögren’s syndrome might be caused by low levels of dehydroepiandrosterone prohormone [48].

The levels of fatty-acid binding protein (FABP) were significantly lower in patients compared to controls in this study. This protein family has recently been suggested as a novel marker for the diagnosis of diseases associated with oxidative stress, such as heart diseases, renal failure, Sjögren’s syndrome and Alzheimer’s disease [49,50,51,52,53]. FABP are intracellular lipid-binding proteins, which exhibit a variety of isoforms depending on the specific organ or cell type. Up to date, at least nine different isoforms have been identified. FABP1 has been suggested as a marker of renal failure, FABP3 as a marker of myocardial infarction, and FABP5 has lately been debated as a diagnostic marker for Sjögren’s syndrome [49,50,51,52]. FABP5 was also shown to be highly expressed in nociceptive dorsal root ganglia neurons, and FABP inhibitors exert analgesic properties on a peripheral and supraspinal level. This indicates that peripheral FABP inhibitors may be used therapeutically to reduce pain and inflammation [54]. Another interesting observation is the significantly lower level of S100-A8, also known as MRP8, in patients compared to controls. This calcium- and zinc-binding protein plays a prominent role in the regulation of inflammatory processes and immune response. It has been demonstrated that S100A8 levels are increased locally in sites of inflammation as well as in the general circulation in rheumatoid arthritis patients. Moreover, the concentration of the protein seems to be strongly associated with disease activity [55].

The present study has some limitations and strengths. A limitation is that the study was performed in adults between 18 and 40 years of age, representing the peak of TMD prevalence, and in only a small number of individuals. Moreover, female participants represented the majority of the study population, mirroring the distribution in the clinic where women prevalently seek care for TMD myalgia. Therefore, sex differences could not be properly addressed. A strength of this study is that the diurnal variation of substances was taken into consideration, since all samples were collected in the morning hours. Saliva collection was also standardized and followed a specific protocol in order to decrease the inter- and intra-individual variability. Finally, the study population was properly examined to exclude systemic or oral conditions that may affect the salivary composition and candidate protein levels.

Further studies are needed to evaluate these potential candidate proteins in a subset of TMD patients. With additional studies to further validate their clinical value in larger patient samples, we may be able to combine these potential candidate proteins with other clinical features to better understand and diagnose TMD myalgia as well as its subclasses and evaluate therapeutic outcomes. 

In conclusion, for the first time, gel-based proteomics was applied to study the salivary protein expression profile of TMD myalgia patients. The analysis showed that there are significant changes in the saliva proteome of TMD myalgia patients compared to healthy controls, with altered levels of immune, metabolic, and stress-related proteins. Significant differences in some proteins and clinical parameters could be observed between subclasses of TMD myalgia (myalgia and myofascial pain), indicating that they display different etiopathogenesis. However, none of the candidate proteins showed statistical correlation with the clinical findings. Further larger studies are needed to evaluate any potential clinical correlation between the candidate proteins and clinical features. 

## 4. Methods and Materials

### 4.1. Participants

In total, 20 patients (28.1 ± 8.8 years of age) referred to the Specialist Clinic for Orofacial Pain and Jaw function, University Dental Clinic, Karolinska Institutet, Huddinge, Sweden, were consecutively enrolled in the study. Twenty pain-free healthy individuals with similar age (28.3 ± 8.4 years), gender, and demographic characteristics were included as controls. Medical history and clinical dental examination for each participant were carefully recorded to evaluate the inclusion and exclusion criteria for each participant. 

Inclusion criteria for the patients were a diagnosis of myalgia (*n* = 10) or myofascial pain with or without referral (*n* = 10) according to the recent DC/TMD Axis I, with at least three months of duration and an average pain intensity during the last 30 days ≥ 3/10 on a 0–10 NRS. Exclusion criteria for both groups were any conditions that could influence pain sensitivity, such as chronic widespread pain, systemic inflammatory disease, whiplash-associated disorder, neurological disorders, pain of dental origin, pregnancy or lactation, and high blood pressure. Patients taking medications that could interfere with the analysis or that could interfere with pain perception or sensitivity, such as anticoagulant treatment and analgesic, antidepressant, or anticonvulsant drugs were also excluded. In addition, patients with factors that could influence saliva collection and composition, such as hypo-salivation, salivary gland diseases, poor oral hygiene, regular tobacco usage, several missing teeth, extensive prosthodontics rehabilitations, oral diseases, and mucosal lesions were excluded from further involvement in the study. One dentist (HJ) calibrated to a reference standard researcher (ME) according to the most recent DC/TMD criteria examined all the patients and controls to ensure they fulfilled all the terms. 

The study was approved (12 March 2014) by the Regional Ethical Review Board in Stockholm, Sweden, (17–31 March 2014) and followed the guidelines of the Declaration of Helsinki. Patients and healthy controls received written and verbal information about the study and signed a consent form prior to sample collection. The study was conducted at the Department of Dental Medicine, Karolinska Institutet, Huddinge, Sweden.

### 4.2. Questionnaires and Clinical Measurements

Participants were asked to complete validated questionnaires and NRS included in the DC/TMD Axis II. The GCPS, PHQ-9, PHQ-15, GAD-7, PCS, PSS-10, JFLS, and ISI [6] were used to assess pain-related physical functioning, symptoms of depression, somatization, anxiety, pain catastrophizing, perceived stress, jaw function, and sleep disturbance. In addition, the participants estimated their physical activity level per week with the alternatives: 1–2 times/month; 1–2 times/week or ≥3 times/week. Current pain intensity on the day of sample collection was assessed on an NRS. The scale ranged from 0 to 10, where 0 indicates “no pain at all”, and 10 indicates “worst imaginable pain”. The CPI was also assessed with the first three question of the GCPS. The CPI was calculated as the average of the current pain intensity and the average and worst pain intensity during the past month. The score was then multiplied by 10 to yield a 0–100 final score [6].

Patients and controls were clinically examined according to the DC/TMD examination, including range of mandibular movements and pain on movements, presence of joint sounds, and palpatory pain of the temporomandibular joint, the temporalis, and the masseter muscles.

### 4.3. Pressure Pain Threshold

The PPT was recorded by an electronic pressure algometer (Somedic Sales AB, Hörby, Sweden). The PPT was recorded at the most prominent point of the masseter muscle and over a reference point on the tip of the index finger on the same side. The procedure was first described to the participant and practiced once to accustom the participant to the procedure. The PPT was then recorded three times at each location. For the analyses, the average threshold of the three recordings was used.

### 4.4. Sample Collection and Preparation

Stimulated whole saliva was collected in the morning using a standardized protocol, as described previously [22,23]. In order to prevent any contamination from other sources, the participants were asked to rinse their mouth with water before saliva collection. Saliva was stimulated with paraffin gum (Orion Diagnostica, Esbo, Finland). After 60 s of chewing, the participants were asked to swallow the saliva present in the mouth and then started to chew and expectorate the saliva into precooled polypropylene tubes coated with protease inhibitor (Sigma Aldrich *v*/*v* 1:500, Saint Louis, MO, USA), until 5 mL of whole stimulated saliva was collected. 

Once collected, the saliva samples were immediately centrifuged at 2500× *g* for 15 min to separate the supernatant from cell pellet and debris. The supernatant was then aliquoted and stored at −70 °C until analysis. 

### 4.5. Gel Electrophoresis

Saliva samples were desalted with 12 mM ammonium bicarbonate and concentrated using Amicon^®^ Ultra-centrifugal filters (Merck Millipore, Billericia, MA, USA). Samples were lyophilized and resolved with 200 µl of 2-DE urea sample buffer according to Gorg et al. [56]. The total protein amounts of the prepared saliva was determined using 2D-Quant kit protein assay according to the manufacturer’s protocol (GE Healthcare UK Ltd., Little Chalfont, UK). From each sample, 300 μg of protein was applied by in-gel rehydration, according to the manufacturer’s instructions, for 10 h using a low voltage (30 V) at pH 3–10 on non-linear 24 cm immobilized pH gradient (IPG) strips (GE Healthcare, Stockholm, Sweden). The proteins were then focused for up to 40,000 Vhs at a maximum voltage of 8000 V to assure a steady state. The IPG strips were immediately stored at −70 °C until analyzed. The IPG gel strips were equilibrated in SDS equilibration buffer (urea 6 M, SDS 4% (*w*/*v*), glycerol 30.5% (*w*/*v*), and Trizma-HCl 50 mM) and dithiothreitol 1% (*w*/*v*) for 15 min and then with iodoacetamide 4.5% (*w*/*v*) for additional 15 min. The second dimension (SDS-PAGE) was carried out using a vertical 2-DE setup (ETTAN™ DALTsix Electrophoresis system, Amersham, Pharmacia Biotech, Uppsala, Sweden), as previously described [18]. Briefly, the IPG strips were mounted on precast homogenous polyacrylamide gels (DALT gel 260 × 200 × 1,0 mm, 12.5%) and run according to the protocol for about 7–8 h (2.5 W per gel, 600 V, 400 mA for 30 min, followed by an additional 5 h at 15 W per gel, until the blue front reached the bottom of the gel) at a constant temperature of 25 °C.

### 4.6. Staining and Image Analysis

The analytical gels were fluorescently stained with One-Step Lumitein™ protein gel stain (Biotium, Hayward, CA, USA) according to the manufacturer’s protocol. After SDS-PAGE, the gels were fixed using 40% methanol and 10% acetic acid solution overnight and then incubated in 350 mL of Lumitein™ protein gel statin solution overnight. The gels were washed and placed in deionized water. The stained gels were then scanned using a charge-coupled device camera system, VersaDoc™ MP 4000 (Bio-Rad Hercules, CA, USA), in combination with a computerized imaging 16-bit system designed for the evaluation of 2-DE patterns (PDQuest V 8.0.1; Bio-Rad Laboratories, Hercules, CA, USA). Protein spots were detected and matched among different samples, the amount of protein in an individual spot was assessed as background-corrected optical density, integrated over all pixels in the spot, and expressed as integrated optical density (IOD).

### 4.7. Protein Identification by LC–MS/MS

The protein spots of interest were excised from the gel and digested with trypsin (Promega Corporation, Madison, WI, USA), as described previously [57]. The trypsinized peptides were analyzed using a nano-liquid chromatography system (EASY-nLC, Thermo Scientific, Waltham, MA, USA) coupled to the LTQ Orbitrap Velos Pro MS (Thermo Scientific). Database searching was performed using the software MaxQuant (version 1.5.8.3) against the human Swissprot/UniProt database with the following parameters: trypsin as digestion enzyme, maximum number of missed cleavages 2, minimum peptide length 6, minimum of 1 unique peptide, parent ion mass tolerance 4.5 ppm, fragment ion mass tolerance 0.5 Da. Fixed modification was set as carbamidomethylation of cysteine, oxidation of methionine as variable modifications and N-terminal acetylation. Protein false discovery rate was set to <1%.

### 4.8. Statistical Analysis

The Shapiro–Wilk’s test was used to test for normality in each distribution. For continuous variables with normal distribution, independent t-test was used. For categorical variable or variables that were non-normally distributed, Mann–Whitney U-test was applied to study differences between groups. Correlations between variables were tested with Spearman correlation test adjusted for multiple comparison according to Bonferroni. Descriptive data are shown as mean (±SD) or median (IQR). For all analyses, the significance level was set to *p* < 0.05. Statistica version 13 (StatSoft, Tulsa, OK, USA) was used.

PCA and OPLS-DA were applied to identify multivariate correlations between proteins and group membership, using SIMCA-*p*+ v.15.0 (UMETRICS, Umeå, Sweden), as described earlier [16], and in accordance with Wheelock and Wheelock [58]. First, PCA, that is an unsupervised method, was used to check multivariate outliers. In the second step, OPLS-DA was applied to investigate the multivariate correlations between proteins and group membership. The VIP indicates the relevance of each X-variable pooled over all dimensions and the Y-variables—the group of variables that best explain Y. VIP > 1.5 was considered significant. R^2^ describes the goodness of fit—the fraction of sum of squares of all the variables explained by a principal component. Q^2^ describes the goodness of prediction—the fraction of the total variation of the variables that can be predicted by a principal component using cross validation methods. R^2^ should not be considerably higher than Q^2^. To validate the model, obtained CV-ANOVA was used. The OPLS-DA model was considered of significant importance if the CV-ANOVA had a *p*-value < 0.05.

For the analysis of protein networks and known involvement in biological processes of the significant proteins, STRING was used. Protein accession numbers (UniProt) for the significant proteins from the OPLS-DA regression were entered in the search engine (multiple proteins) with the following parameters: organism was *Homo sapiens*, maximum number of interactions was query proteins only, interaction score was set to minimum required interaction score of medium confidence (0.400), and FDR ≤ 0.05 was used when classifying the Biological Process (GO) of each protein. For the obtained network, the PPI enrichment *p*-value was reported.

## Figures and Tables

**Table 1:** **Descriptive data.** Demographic features of patients with temporomandibular disorders myalgia (*n* = 20) and healthy controls (*n* = 20). Questionnaire scores are presented as mean ± standard deviation or as median (interquartile range). Statistical parameters are reported only when the distributions in the two groups differed significantly, *p* < 0.05 (t-test or Mann–Whitney U-test).

Variable	Patients	Controls	*p*-Value
**Age (years)**	28.1 ± 8.8	28.3 ± 8.4	*p* > 0.05
**Sex, (n, F/M)**	14/6	14/6	*p* > 0.05
**Body Mass Index (kg/m^2^)**	24.4 ± 3.8	22.7 ± 3.0	*p* > 0.05
**Number of teeth**	28 (2)	30 (3)	*p* > 0.05
**Pain-free opening (mm)**	41.4 ± 10.8	56.4 ± 5.9	*p* < 0.0001
**Maximum unassisted opening (mm)**	54.0 ± 6.0	57.7 ± 6.1	*p* > 0.05
**Salivary Flow (ml/min)**	1.57 ± 0.50	1.74 ± 1.05	*p* > 0.05
**Pain duration (years)**	6.3 ± 6.3	0 (0)	*p* < 0.001
**Current pain intensity (NRS)**	6 (2.5)	0 (0)	*p* < 0.001
**CPI**	65 (27)	0 (0)	*p* < 0.001
**PHQ-9 Score (0–36)**	6.5 (7)	1 (3.5)	*p* < 0.001
**PHQ-15 Score (0–30)**	11.5 (9)	2.5 (4)	*p* < 0.0001
**GAD-7 Score (0–28)**	3.5 (8.5)	1 (2.5)	*p* < 0.01
**PSS-10 Score (0–40)**	15.5 (10)	10 (8)	*p* < 0.01
**JFLS Score (0–10)**	1.65 (2.0)	0 (0)	*p* < 0.0001
**PCS Score**	15 (18)	5 (10)	*p* < 0.01
**ISI Score**	10 (15)	5 (5)	*p* < 0.01
**PPT reference (kPa)**	382 ± 127	437 ±130	*p* > 0.05
**PPT masseter muscle (kPa)**	179 ± 63	272 ± 81	*p* < 0.001

*n* = number of subjects in each group; NRS = Numeric Rating Scale; CPI = Characteristic Pain Intensity; PHQ = Patient Health Questionnaire; GAD = Generalized Anxiety Disorder; PSS = Perceived Stress Scale; JFLS = Jaw Functional Limitation Scale; PCS = Pain Catastrophizing Scale; ISI = Insomnia Severity Index; PPT = Pressure Pain Threshold.

**Table 2:** **Proteins altered in the saliva from patients compared to controls.** Identified salivary proteins that were altered in patients (Pat) with temporomandibular disorder myalgia compared to healthy controls (Con). Proteins with a VIP above 1.5 in the orthogonal partial least-squares discriminant analysis model are shown. The *p*-value is according to the Mann–Whitney data analysis. Arrows ↑ and ↓ indicate up- and downregulated proteins in patients compared to controls.

Spot No	Protein Name	UniProt ID	VIP	*p*-Value	Pat vs Con
**211**	Immunoglobulin J chain	P01591	2.026	0.005	↓
**9502**	Phosphoglycerate kinase 1	P00558	1.959	0.056	↑
**7202**	Glyceraldehyde-3-phosphate dehydrogenase	P04406	1.944	0.04	↑
**2102**	Fatty acid-binding protein	Q01469	1.823	0.042	↓
**6202**	Immunoglobulin kappa light chain	P0DOX7	1.791	0.04	↑
**4801**	Alpha-amylase 1; Alpha-amylase 2B	P04745/P19961	1.783	0.213	↑
**2501**	Alpha-amylase 1; Alpha-amylase 2B	P04745/P19961	1.756	0.007	↓
**9205**	Cysteine-rich secretory protein 3	P54108	1.745	0.053	↑
**1602**	Zinc-alpha-2-glycoprotein	P25311	1.738	0.026	↑
**9404**	Chitinase-3-like protein 2	Q15782	1.713	0.033	↑
**5401**	Alpha-amylase 1; Alpha-amylase 2B	P04745/P19961	1.689	0.06	↑
**5501**	Alpha-amylase 1; Alpha-amylase 2B	P04745/P19961	1.674	0.027	↑
**209**	Interleukin-1 receptor antagonist protein	P18510	1.639	0.025	↑
**3601**	Alpha-amylase 1; Alpha-amylase 2B	P04745/P19961	1.630	0.168	↓
**8001**	Protein S100-A8	P05109	1.622	0.004	↓
**1202**	Albumin (N terminal fragment)	P02768	1.572	0.285	↑
**212**	Immunoglobulin J chain	P01591	1.569	0.009	↓
**5603**	Alpha-amylase 1; Alpha-amylase 2B	P04745/P19961	1.530	0.172	↑
**12**	Thioredoxin	P10599	1.523	0.176	↓
**210**	Immunoglobulin J chain	P01591	1.519	0.028	↓

**Table 3:** **Subclasses of temporomandibular disorders.** Differences between patients diagnosed with myalgia (*n* = 10) and patients diagnosed with myofascial pain with or without referral (*n* = 10) according to the Diagnostic criteria for temporomandibular disorders. Orthogonal projections to latent structure model characteristics: *R*^2^ = 0.7, *Q*^2^ = 0.4, CV-ANOVA = 0.01. Variables with VIP above 1.0 in the orthogonal partial least-squares discriminant analysis model are shown in decreasing order of VIP values. The *p*-value is according to the Mann–Whitney data analysis.

Variable	Myalgia	Myofascial Pain	VIP	*p*-Value
**Phosphoglycerate kinase 1**	1 282 ± 519	323 ± 441	2.090	0.001
**PPT masseter muscle (kPa)**	227 ± 59	141 ± 31	1.945	0.001
Level of physical activity *	≥ 3 times/week	1–2 times/week	1.639	0.039
**PHQ-9 SCORE (0–36)**	4 (6)	8 (8)	1.571	0.121
**Alpha-amylase 1; Alpha-amylase 2B**	2 927 ± 1 885	1 102 ± 1 624	1.569	0.017
**Current pain intensity (NRS)**	4 (3)	6 (1)	1.511	0.023
**CPI**	53 (20)	73 (17)	1.474	0.023
**GCPS (Grade 0–IV)**	2 (2)	3 (1)	1.431	0.131
**Alpha-amylase 1; Alpha-amylase 2B**	1 785 ± 1 498	845 ± 827	1.389	0.140
**Chitinase-3-like protein 2**	1 679 ± 1 177	675 ± 636	1.265	0.026
**Glyceraldehyde-3-phosphate dehydrogenase**	5 862 ± 4 225	2 568 ± 4 822	1.260	0.011
**PHQ-15 Score (0–30)**	8 (11)	12 (5)	1.210	0.273
**PPT reference (kPa)**	419 ± 151	353 ± 103	1.210	0.450
**ISI Score**	9 (13)	12 (14)	1.188	0.488
**Headache duration (years)**	3 ± 4	8 ± 4	1.120	0.037
**PSS Score (0–40)**	13 (11)	19 (7)	1.073	0.121

PPT = Pressure Pain Threshold; GCPS = Graded Chronic Pain Scale; * Median level of physical activity/week.
